# Th17 Cells Pathways in Multiple Sclerosis and Neuromyelitis Optica Spectrum Disorders: Pathophysiological and Therapeutic Implications

**DOI:** 10.1155/2016/5314541

**Published:** 2016-01-28

**Authors:** Giordani Rodrigues Dos Passos, Douglas Kazutoshi Sato, Jefferson Becker, Kazuo Fujihara

**Affiliations:** ^1^Brain Institute and Department of Neurology, Faculty of Medicine, Pontifical Catholic University of Rio Grande do Sul (PUCRS), Avenida Ipiranga 6690, 90610-000 Porto Alegre, RS, Brazil; ^2^Department of Neurology and LIM-15, Faculty of Medicine, University of Sao Paulo (USP), Avenida Dr. Arnaldo 455, 01246-903 São Paulo, SP, Brazil; ^3^Department of Multiple Sclerosis Therapeutics, Tohoku University Graduate School of Medicine, 1-1 Seiryo-machi, Aobaku, Sendai, Miyagi 980-8574, Japan

## Abstract

Several animal and human studies have implicated CD4+ T helper 17 (Th17) cells and their downstream pathways in the pathogenesis of central nervous system (CNS) autoimmunity in multiple sclerosis (MS) and neuromyelitis optica spectrum disorders (NMOSD), challenging the traditional Th1-Th2 paradigm. Th17 cells can efficiently cross the blood-brain barrier using alternate ways from Th1 cells, promote its disruption, and induce the activation of other inflammatory cells in the CNS. A number of environmental factors modulate the activity of Th17 pathways, so changes in the diet, exposure to infections, and other environmental factors can potentially change the risk of development of autoimmunity. Currently, new drugs targeting specific points of the Th17 pathways are already being tested in clinical trials and provide basis for the development of biomarkers to monitor disease activity. Herein, we review the key findings supporting the relevance of the Th17 pathways in the pathogenesis of MS and NMOSD, as well as their potential role as therapeutic targets in the treatment of immune-mediated CNS disorders.

## 1. Introduction

Multiple sclerosis (MS) is a chronic immune-mediated demyelinating disease of the central nervous system (CNS) characterized by a relapsing-remitting (RR) or a progressive course with multifocal CNS dysfunctions [1]. Neuromyelitis optica spectrum disorders (NMOSD) include the entity previously known as neuromyelitis optica (NMO) and patients with limited forms (e.g., only myelitis or optic neuritis) and comprise a phenotypic continuum of primarily immune-mediated astrocyte injury, rather than a primary demyelinating disease, with preferential involvement of the optic nerves, brainstem, and the spinal cord [2, 3].

The nosology of NMO remained controversial for more than one century after its first description, by Devic, in 1894 [3]. It was speculated that it could represent a topographically restricted severe MS variant. A considerable advance in the understanding of those disorders was the identification of pathogenic autoantibodies against aquaporin-4 (anti-AQP4-IgG) in patients with NMO, which allowed for the establishment of NMO as a distinct nosological entity [3]. Despite the fact that both diseases have an inflammatory process restricted to the CNS and a relapsing course in the majority of patients, there are major differences in clinical definition and understanding of the two diseases. Astrocyte injury leading to secondary demyelination is the hallmark of NMO, at least in those patients who are AQP4-IgG-seropositive, while primary demyelinating lesions with T cell and macrophage infiltration are seen in MS [2]. From the clinical and radiological standpoint, both disorders may present optic neuritis, transverse myelitis, and/or demyelinating brain lesions, but some features are specially suggestive of NMO, such as bilateral optic neuritis, involvement of the optic chiasm, or severe residual visual loss; a complete transverse myelitis, usually with longitudinally extensive lesions on the MRI; and an area postrema syndrome, characterized by intractable nausea, vomiting, and hiccups [3]. Besides that, it has been shown that several immunological therapies commonly used for MS fail to control or even increase disease activity in NMOSD [4], thus suggesting a distinct underlying pathophysiological process in each of those disorders and highlighting the need for a precise distinction between them in order to avoid the potentially harmful consequences of a misdiagnosis.

In both MS and NMOSD, T-B cell interaction has been pointed out as an important factor in the genesis of the disease process. In especially MS, increasing therapeutic options became available in recent years, and some of them involve control of autoreactive T cells, which highlights the importance of further understanding of the role of each of those cell types. Some knowledge about immune mechanisms involving autoreactive T cells comes from experimental autoimmune encephalomyelitis (EAE), the animal model of MS, and from animal models using passive human anti-AQP4-IgG transfer in NMO.

Initially, the group of CD4+ T lymphocytes known as helper T (Th) cells was believed to differentiate into two mutually exclusive phenotypes: type 1 ones (Th1), which are classically induced by interleukin- (IL-) 12 and produce interferon gamma (IFN-*γ*), and type 2 ones (Th2), which are stimulated and secrete IL-4 [5]. At that time, the Th1 pathway, regarded as proinflammatory, was considered to be the most important mediator of the pathogenesis of both EAE and MS, while the Th2 pathway would have an antagonist effect on Th1 cells and, consequently, a beneficial effect on the disease process [6]. However, subsequent studies provided consistent evidence that mice were still susceptible to EAE after genetic ablation of key cytokines of the Th1 pathway, such as IFN-*γ*, indicating that other unknown pathways were involved [7].

More recently, a new phenotype of Th cells was described, namely, Th17, whose signature cytokine is the IL-17 family. Th17 cells have been implicated in several autoimmune disorders and these cells seem to be relevant in the development of CNS autoimmunity. Here, we review the key findings from animal and human studies supporting the role of Th17 pathways in the MS and NMOSD pathogenesis and potential therapeutic targets under clinical investigation.

## 2. Brief Review of the Th17 Pathways

In 2005, it was demonstrated that näive Th cells would differentiate into a new lineage called Th17, due to its capacity to produce large amounts of IL-17 [8, 9]. Currently, the cytokine previously named as IL-17 is referred to as IL-17A, since it became clear that IL-17 actually represents a family of cytokines, which includes IL-17A to IL-17F.

The process of differentiation of näive Th cells into Th17 is dependent on IL-23 [10] and potently inhibited by IFN-*γ* and IL-4 [8]. IL-23 knockout mice are resistant to EAE and lacked Th17 cells [11], suggesting that the Th17 pathway is implicated in the pathogenesis of EAE. However, the differentiation of T näive cells into Th17 cells may be induced not only by IL-23, but also by the combination of transforming growth factor beta 1 (TGF-*β*1) and IL-6. Moreover, IL-1*β*, in combination with IL-6 and IL-23 and independently of TGF-*β*1, may induce a different (and pathogenic) phenotype of Th17 cells, characterized by the coexpression of RAR-related orphan receptor gamma t (ROR*γ*t) and T-bet [12].

Indeed, Th17 cells can present different phenotypes, pathogenic or not, according to the modulating factors they are exposed to, such as IL-23, and the differential expression of some cytokines and chemokines, such as IL-17A, IL-17F, IL-21, and ROR*γ*t transcription factor [13]. Moreover, Th17 cells have impressive plasticity, that is, ability to transition between different phenotypes throughout their life span [7]. It has been demonstrated that a significant proportion of IL-17-producing (Th17) cells converts into IFN-*γ*-producing T cells, partially due to IL-23-mediated reprogramming [14]. There is also a subset of Th17 cells that, in addition to IL-17A, simultaneously express IFN-*γ* and have chemokine receptors from both Th17 and Th1 cells [15].

Granulocyte macrophage colony stimulating factor (GM-CSF) is a growth factor that acts as a proinflammatory cytokine and is critically involved in Th17 and other cell-mediated immune responses. It is produced by several different cells, especially T cells, in response to IL-23 and IL-1*β* [16] and induces the activation, maturation, and differentiation of macrophages and of dendritic cells (which secrete IL-23 and IL-6) [17]. Notably, there is a positive feedback loop between GM-CSF and IL-23, which plays a critical role in the expansion of pathogenic Th17 cells. Indeed, studies with EAE have shown that GM-CSF is essential for mediating Th17 cells-induced encephalitogenicity [16, 18, 19]. Recently, another study with EAE has suggested that the GM-CSF-producing T cells likely represent a distinct subset of T helper cells, designated as Th-GM [20].

Currently, after several studies indicating that the IL-17 family plays a crucial role in the development of EAE [21, 22], the pathogenic potential of Th17 pathways, in addition to that of Th1 pathways, in the development of EAE has been widely accepted, although not fully understood. Th1-induced EAE presents the classical phenotype, characterized by an ascending caudocranial paralysis, while Th17 polarization induces EAE with an atypical phenotype, characterized by ataxic gait [23].

Based on the previously mentioned findings, the classical Th1 paradigm was replaced by the Th1/Th17 paradigm in both EAE and MS. It is postulated that Th17 cells would play a more relevant role in the initial phases of EAE and MS, while Th1 cells would be more important in later stages of the inflammation in the CNS [6].

## 3. Modulation of Th17 Responses by Environmental Factors

Relevant modulation of Th17 responses occurs in mucosal tissues, especially those of the lungs [24] and gastrointestinal tract [25]. This modulation is dependent on the complex interaction of local immune elements with a multitude of pathogens, nutritional components, and other environmental factors. It is believed that T cell clones stimulated by these interactions in the periphery would induce activated T cells able to migrate through the blood-brain barrier (BBB) and induce damage in the CNS. Further studies are still required to better understand the role of environmental factors in MS and other autoimmune diseases, but this growing amount of evidence can provide new targets for therapeutic interventions.

One of the mechanisms of interaction between environmental factors and T cells is the aryl hydrocarbon receptor (AHR), which modulates the differentiation towards either Th17 or regulatory T (Treg) cells, increasing or decreasing, respectively, the severity of EAE, depending on its ligand [26]. Different compounds from the environment, including commensal microbiota and human pathogens, can act as a ligand for the AHR; moreover, such compounds may act only locally (i.e., in the mucosal tissues) or reach the circulation, causing changes in the immune system of different compartments [7].

Traditionally, systemic infections (especially by viral agents) have been believed to play a role in triggering or modulating the immune process that ultimately may lead to autoimmune CNS diseases. More recently, however, several studies have pointed out that, under special circumstances, normal intestinal microbiota may also activate previously quiescent autoreactive T cells in the gut-associated lymphoid tissues (GALT), thus precipitating autoimmunity [25]. There is some evidence implicating the small intestine as the major site for activation of effector Th1 and Th17 cells and segmented filamentous bacteria as the major inducers of IL-17-producing immune cells [27, 28]. As an example, cell cultures derived from NMO patients showed a higher Th17 responsiveness to* Escherichia coli*, associated with elevated IL-1*β*, IL-6, and IL-17 production and decreased IL-10 release, when compared to healthy controls [29]. Besides the complex regulation by cytokines and commensal or pathogenic microorganisms in T cell differentiation and function, Th cells are also regulated (and can be dynamically reprogrammed) by cellular metabolic pathways, including those related to glucose, amino acid, and lipid metabolism [30, 31]. Further studies are required to investigate the role of gut microbiota in the pathogenesis of MS and NMO.

Moreover, high concentrations of sodium chloride and high dietary salt intake have also been shown to enhance the differentiation of Th17 cells and lead to a more severe form of EAE [32]. That phenomenon seems to be mediated by serum glucocorticoid kinase 1 (SGK1), a downstream molecule of IL-23 signaling. SGK1 expression increases after elevation of salt concentration, promoting IL-23R expression and subsequently enhanced Th17 cell differentiation and autoimmunity development [33]. Interestingly, a recently published prospective study has found a positive correlation between higher sodium intake and increased disease activity, measured by both clinical and radiological parameters in patients with MS [34]. Further studies are required to evaluate if the large amounts of salt intake could increase the risk of developing MS.

Finally, substance P, a stress-related neuropeptide, was shown to influence T cell and cytokine profiles in cell cultures derived from patients with generalized anxiety disorder [35]. Complex T cell functional dysregulation (including Th1 and Th2 deficiency and Th17 hyperactivation) was further enhanced by substance P, thus suggesting rationale for the influence of chronic stress and anxiety disorders in individuals' susceptibility to autoimmune disease [35]. However, such influence of substance P in immune function has not been studied in MS or NMOSD yet.

## 4. Th17 as Pioneering Cells in the Breakdown of the BBB

After modulation and selection of T cells in peripheral tissues, pathogenic autoreactive T cells need to cross the BBB in order to cause inflammation into the CNS. Th17 cells have a large number of chemokines and chemokine receptors required to cross the BBB, which enables them to disrupt the BBB and access the CNS via several different pathways.* In vitro* and* in vivo* studies have shown that, through the action of IL-17A and IL-22, Th17 cells can efficiently disrupt BBB tight junctions, express high levels of the cytolytic enzyme granzyme B, and promote the recruitment of additional CD4+ lymphocytes from the systemic circulation into the CNS [36]. Th17 cells are also able to induce CXCL1 and CXCL2, chemokines that are potent attractants for polymorphonuclear cells and play an important role in the breakdown of the BBB in EAE [37]. Th17 cells can access the subarachnoid space via upregulation of the CCR6 receptor, expressed in the epithelium of the choroid plexus, in a process that is critical for the initiation of EAE [38]. Moreover, IL-17A is a key factor in the breakdown of the BBB by direct impairment of its integrity due to the formation of reactive oxygen species within the endothelial cells [39]. Evidences from EAE demonstrate that Th1 cells preferentially access the CNS by using the *α*4*β*1 integrin, while Th17 cells do that by means of the *α*L*β*2 integrin (LFA-1) [40]. Melanoma cell adhesion molecule (MCAM) or CD146 is another adhesive molecule expressed by Th17 cells, but not by Th1 cells [41].

Some T cells can secrete both IL-17 and IFN-*γ*, being called Th1/Th17 cells. These cells can infiltrate the CNS early in the course of EAE and may be involved in microglial activation and thus may have an important role in the development of the disease [42]. In addition, increased proportions of Th1/Th17 cells were found in the blood and in the brain tissue of MS patients [15]. This amount of evidence suggests that Th17 cells act as pioneering cells in the induction phase of EAE and, presumably, in the early phases of MS.

## 5. Th17 in MS

Following studies that suggested the role of Th17 responses in EAE, several findings from different groups using different techniques have provided substantial evidence that the Th17 pathways play a critical role in MS (a summary of the most important studies provided in Table 1).

An increased frequency of Th17 cells is detected in the peripheral blood and cerebrospinal fluid (CSF) of some RRMS and clinically isolated syndrome (CIS) patients, especially during the acute episode, when compared to patients with noninflammatory neurological diseases [43–47]. Increased proportion of Th17 cells, as well as increased levels of IL-17A (protein and messenger RNA [mRNA]), was observed in the brain tissue of MS patients, especially in acute and chronic active lesions, compared to healthy controls [48]. Th17 cells may also have a role in progressive forms of MS [49]. Finally, levels of GM-CSF, which is essential for Th17 responses as discussed earlier in this review, were also shown to be elevated in the CSF of MS patients [50, 51] and in the blood of MS (but not of NMO) patients [52].

Th17-related molecules were shown to correlate with parameters of disease activity in MS.* In vitro* studies demonstrated that the amount of IL-17 (and also IL-5) produced by mononuclear cell cultures from patients with MS after stimulation with human myelin basic protein correlates with the number of active lesions on magnetic resonance imaging (MRI) [53]. The proportion of Th17 cells, their subset effector memory Th17 cells (CD4+/CD45RO+/CCR7−), and the level of IL-17A correlated with disease severity as measured by the Expanded Disability Status Scale (EDSS), while the proportion of another subset, central memory Th17 cells (CD4+/CD45RO+/CCR7+), correlated with relapse frequency, in both MS and NMO [54]. Serum IL-17F (but not IL-17A) correlated with the number of MS relapses in two years [47].

## 6. Th17 in NMOSD and Its Animal Models

Much of the evidence regarding pathogenesis of NMOSD comes from studies on opticospinal MS (OSMS) and NMO. The former is no longer considered to be a variant of MS, since most of those patients actually had NMO [3]; hence OSMS is considered as an obsolete term.

Although the important role of B cell autoimmunity against aquaporin-4 (AQP4), by means of the anti-AQP4 immunoglobulin G (IgG), a T cell dependent immunoglobulin type (IgG1), in mediating CNS lesions is clearly established, many aspects of tissue damage in NMOSD remain poorly understood [55]. However, T cell-related mechanisms have been increasingly implicated in NMOSD [56–58]. AQP4-specific T cell responses were demonstrated to be amplified in NMO patients, whose T cells were shown to exhibit a Th17 polarization, partially mediated by increased production of IL-6 [59].

In some studies, IL-17 was increased in the CSF [60, 61] and in the blood [54] of NMO patients. Another study on cytokines in NMO did not find an increase in serum or CSF levels of IL-17 but did find an increased level of other Th17-related cytokines, notably IL-6 [52], which is a proinflammatory cytokine that increases the survival of plasmablasts capable of producing anti-AQP4-IgG and is also involved in the development of Th17 cells, which can also support B cell development and induce further tissue injury [62].

Th17-related markers have also been shown to correlate with parameters of disease activity and severity in NMOSD. The release of IL-6 and IL-21 by polyclonally activated CD4+ T cells derived from NMO patients was demonstrated to correlate directly with neurological disability [63] and* in vivo *and* in vitro* levels of IL-6 were higher among NMO patients who experienced relapse within a 2-year follow-up [64]. CSF levels of both IL-17A and the downstream cytokine IL-8 were found to have a positive correlation with spinal cord lesion length in NMO [60]. As previously mentioned in studies with MS, the proportion of effector memory Th17 cells and IL-17A levels correlated with EDSS, and the proportion of central memory Th17 cells correlated with relapse frequency in NMO [54].

The development of NMO-like disease models in animals has provided important insights into the pathogenesis of NMOSD. After the pathogenic potential of anti-AQP4-IgG has been demonstrated, it was shown that AQP4-specific T cells could also induce a NMO-like disease in rats, independent of anti-AQP4-IgG [65, 66]. Recently, another NMO-like model was developed in mice using AQP4-reactive T cells polarized into a Th17 phenotype (also independent of anti-AQP4-IgG), promoting lesions in the optic nerve and spinal cord [67].

A summary of the main studies supporting the involvement of Th17-related pathways in NMOSD is provided in Table 2.

## 7. Different Immunologic Profiles in MS and NMOSD

Several studies have already addressed the immunological differences between MS and NMO. However, a clear definition of immunologic profiles that differentiate MS and NMO remains controversial.

Investigators have assessed the levels of several cytokines and chemokines in the serum and/or CSF of patients with NMO and MS and compared them between both diseases and between each of them and a control group composed of patients with noninflammatory neurological disorders [52]. Both Th2- and Th17-related molecules were found to be upregulated in NMO, except, interestingly, for the signature cytokine of each of those pathways, that is, IL-4 and IL-17 family [52]. Of note, among the Th17-related molecules elevated in NMO, IL-6 seemed to be the most relevant one [52]. In the same study, the Treg-related cytokine IL-10 was elevated in both NMO and MS, whereas Th1-related cytokines and molecules were upregulated only in MS [52].

Another study found increased levels of Th1-related markers in NMO when compared to MS, while the levels of Th17-related markers were similar between both diseases [68]. Investigators from the same group found increased proportion of Th17 cells and of IL-17-secreting T CD8+ cells, in both MS and NMO, especially during relapses [69]. The same study did find IL-17 levels, as well as those of IL-23, to be higher in NMO than in MS, leading the authors to speculate whether that would explain the more aggressive nature of NMO when compared to MS [69].

Another study compared NMO, RRMS, and PPMS and reported an increased expression of Th17- and Th1-related cytokines as being characteristic of NMO [70]. Further studies are required to clarify if cytokine levels are useful to indicate disease activity and if interference in the Th17 pathway can reduce inflammation in the CNS during relapses.

## 8. Effects of Current Therapy on the Th17 Axis

Since Th17 responses seem to be relevant in the pathogenesis of MS and NMOSD, great interest has been put in identifying possible effects of the currently available therapeutic agents on the Th17 pathways. A better understanding of those effects and of the different immunologic profiles of MS and NMOSD could potentially provide an explanation to why some NMOSD patients get worse when undergoing treatment with MS-targeted disease-modifying therapies, such as interferons. It could also provide valuable insights into potential mechanisms to be addressed by new or repositioned drugs.

Intravenous methylprednisolone (IVMP) is the most widely used treatment for acute relapses in both MS and NMOSD. A significant reduction in Th17 cell counts, IL-17A and IL-23R production, and RAR-related orphan receptor c (RORc) mRNA expression has been observed in MS patients after IVMP pulse therapy [54, 71]. A reduction in the same markers, except for IL-17A, was also seen in NMO patients after IVMP treatment [54]. Even though Th17 cells as a whole were decreased in both NMO and MS after IVMP therapy, a stratified assessment of their responses according to some of their subsets showed that central memory Th17 and effector memory Th17 cells were decreased only in NMO and not in MS [54]. These differences in the response to corticosteroids may explain the effectiveness of such drugs in NMO to reduce the risk of further attacks [54]. Progressive MS patients undergoing monthly IVMP pulse therapy presented no changes in the phenotype of Th17 cells [72].

Another therapeutic option for acute relapses in MS and NMOSD is intravenous immunoglobulin (IVIg).* In vitro* studies have demonstrated that IVIg inhibits the differentiation and amplification of Th17 cells and the production of IL-17A, IL-17F, IL-21, and CCL20 [73]. The inhibitory effect of IVIg on IL-17A seems to be mediated by the modulation of intracellular signaling pathways and not by passive neutralization by anti-IL-17 antibodies from the IVIg preparations [74].

Several drugs have been developed as disease-modifying therapies for MS, and many of them have been shown to modulate the Th17 axis. One of these immunomodulatory drugs is the recombinant IFN-*β*, which includes IFN-*β* 1a and IFN-*β* 1b. It was suggested that IFN-*β* inhibits the differentiation of Th17 cells in mice and hence modulates the severity of EAE, by acting on the toll-IL-1 receptor domain-containing adaptor inducing IFN-*β*-dependent (TRIF-dependent) type I IFN induction pathway and its downstream signaling pathways, especially by means of an increased production of IL-27 [75]. In both mice with EAE and patients with MS, IFN-*β* also decreased the ability of dendritic cells to stimulate the production of IL-17 by Th17 cells and increased the production of IL-27 by dendritic cells, shifting the proinflammatory response into an anti-inflammatory one [76]. In humans, IFN-*β* therapy was shown to downregulate the expression of IL-1*β*, IL-23R, RORc, and IL-17A and upregulate the expression of IL-12, IL-27, and IL-10, suppressing the differentiation of näive T cells into Th17 cells. These effects may explain some of the IFN-*β*'s immunomodulatory effect in MS [77].

However, IFN-*β* is not always effective in reducing CNS autoimmunity, and efforts have been done to identify factors that could predict the response to IFN-*β* therapy. One study pointed out that mice with Th1-induced EAE did benefit from IFN-*β* treatment, whereas deleterious effects were observed in mice with Th17-induced EAE [78]. Indeed, IFN-*β* seems to be effective in diseases primarily driven by Th1, whereas it has proinflammatory effects in Th2-driven diseases [79]. In a study with RRMS patients, pretreatment levels of IL-17F and of endogenous IFN-*β* were higher in nonresponders than in IFN-*β* treatment responders [78]. A subsequent study, however, did not confirm the role of serum IL-17F in the prediction of poor response to IFN-*β* therapy [80]. Interestingly, IFN-*β* is not effective in reducing relapse rates or preventing disability in patients with NMOSD [81] and may even trigger severe exacerbations in those patients [82, 83]. It has been suggested that the poor response to IFN-*β* in NMOSD may be related to elevated levels of IL-17 [4], which corroborates some of the findings from earlier studies with EAE models and MS patients.

Glatiramer acetate (GA) is another traditional disease-modifying drug for MS. In EAE mice treated with GA, Th17 cells were drastically reduced, while Treg cells were increased [84]. However, further studies are required to clarify if the same effects are observed in MS patients treated with GA. Despite some anecdotal reports of patients with NMOSD who seemed to benefit from GA therapy [85, 86], as well as some speculations regarding the rationale for a potential benefit of GA in NMOSD [87], currently there is no evidence to support this indication, as well as no data regarding its effect on Th17 in NMOSD patients.

In patients with MS, fingolimod (FTY720) reduces Th17 central memory T cells in peripheral blood, presumably due to the retention of those cells in secondary lymphoid organs [88]. Another study pointed out that, after initiation of fingolimod therapy, half of the patients had a reduction in the proportion of circulating Th17 cells, whereas the other half (including the only one patient with relapses in that sample during the follow-up period) had an increase in the proportion of those cells, suggesting that a slower reduction in circulating Th17 cells after fingolimod initiation would predispose to relapses [89]. In NMOSD, however, treatment with fingolimod was reported to trigger extensive brain lesions [90] or a fulminant course [91]; thus it is not recommended for NMOSD. Since eosinophils have been implicated in NMOSD pathogenesis [92] and fingolimod may promote bone marrow egress of eosinophils [93] and other pathogenic proinflammatory cells, that would explain the severe disease activity in NMOSD patients exposed to fingolimod.

Natalizumab is another commonly used treatment for MS. It acts by interfering with lymphocyte migration across the BBB, which is mediated by the interaction between *α*4*β*1 integrin (on the surface of lymphocytes) and vascular-cell adhesion molecule 1 (VCAM-1; an endothelial receptor in CNS vessels) [94]. Specifically, it binds to the integrins, thus preventing them from binding to their endothelial receptors [94]. Even though such process is also required for CNS inflammation to develop in NMOSD, natalizumab was reported to fail in controlling disease activity in patients with NMOSD [95] or even triggering catastrophic exacerbations [96, 97]. Like fingolimod, natalizumab may increase the number of peripheral eosinophils [94], which could account for the increased disease activity in NMOSD patients treated with natalizumab. The specific effects of natalizumab over the Th17 cells have not been described, and given the number of receptors present in such cells, it might be possible that pathogenic Th17 cells can use alternative pathways not requiring binding to *α*4*β*1 integrin to access the CNS.

In MS patients, treatment with dimethyl fumarate inhibited the maturation of dendritic cells and thus the generation of IFN-*γ*-producing (Th1) and IL-17-producing (Th17) cells [98]. No evidence on the use of dimethyl fumarate in NMOSD is available, so the use of this drug is not recommended at this time point.

Finally, treatment of aggressive MS with chemoablation and hematopoietic stem cell transplantation seems to exert its effect by decreased Th17 and Th1/Th17 responses, rather than Th1 pathway responses [99]. Even though some studies reported cases of NMOSD treated with stem cell transplantation [100–103], with variable results, none of them reported its influence on Th17 cells.

Several cytotoxic, immunomodulatory, and B cell depleting therapies are available for prevention of attacks in NMOSD, including prophylactic corticosteroids, plasma exchange, and IVIg, as well as azathioprine, mycophenolate mofetil, methotrexate, mitoxantrone, cyclophosphamide, and rituximab [104]; however, their precise effects on Th17 cells in NMOSD are not known to date. Rituximab (an anti-CD20 monoclonal antibody) and IVIg in combination have been shown to modulate T cell subsets and humoral immune responses in NMOSD [105]. Some data regarding the specific effects of the aforementioned drugs on the Th17 pathways is available from studies on other autoimmune diseases. Rituximab decreases Th17 cell responses in rheumatoid arthritis [106] and the IL-17 production in Sjögren's syndrome [107]. Methotrexate, with or without corticosteroids, reduces Th17 cell frequency [108], normalizes the Th17/Treg balance [109], and suppresses IL-17 production [110] in rheumatoid arthritis.

## 9. Emerging Therapies Targeting the Th17 Pathways

A few monoclonal antibodies targeting different Th17-related cytokines have been tested in MS so far. The first one, ustekinumab, was an antibody against IL12 and IL-23, which are critical for the maintenance of Th17 cells. Nevertheless, in a phase II study, ustekinumab did not show efficacy in the reduction of new enhancing lesions on brain MRI, number of relapses, or change from baseline EDSS after 23 weeks [111].

Secukinumab (AIN457) is a recombinant, highly selective, fully human monoclonal antibody against IL-17A.* In vitro* treatment of human astrocytes with secukinumab was shown to upregulate the levels of IL-6 and to decrease the levels of proinflammatory molecules [112], thus making it suitable for phase II studies in MS. Clinical trials in some other autoimmune diseases yielded promising results, with no significant safety concerns [113], and so far, three phase II trials assessing secukinumab in MS have been started. The first one (ClinicalTrials.gov identifier: NCT01051817) was a randomized, multicenter, double-blind, proof-of-concept study to assess the effect of secukinumab versus placebo on MRI parameters of disease activity over a 24-week period in patients with RRMS [114]. The results have been presented in a conference, as follows: the primary outcomes, number of new gadolinium-enhancing lesions, and number of combined unique active lesions decreased by 67% (*P* = 0.003) and by 49% (*P* = 0.087), respectively, in the secukinumab group; as a secondary outcome, the annualized relapse rate decreased by 43% in the secukinumab group, which was not statistically significant, maybe because the study was not powered to assess that outcome [114]. However, due to methodological issues (especially small sample size and important demographic, clinical, and radiological differences between groups on baseline), caution must be taken in the interpretation of those findings [115]. The second trial (ClinicalTrials.gov identifier: NCT01874340), which is an extension of the first one, has had no results published yet. The third trial (ClinicalTrials.gov identifier: NCT01874340), which is a larger phase II study, was terminated early “based upon development of another anti-IL-17 fully human monoclonal antibody with better potential for treating MS patients,” according to information provided by the sponsor. Actually, the new compound is CJM112, a new fully human anti-IL17A monoclonal antibody, whose phase II trial was not yet registered on https://ClinicalTrials.gov (accessed on 19 September 2015).

Future perspectives include targeting genes and soluble factors that mediate Th17 cell expansion and have been shown to be differentially expressed in MS patients [116], as well as modulating metabolic pathways that are relevant for the regulation of Th17 responses [30, 31].

## 10. Conclusions

Even though not completely understood, the role of Th17 cells in the pathogenesis of both MS and NMOSD is very well established by several findings from studies in humans and animal models. Th17-related pathways seem to be modulated by many of the currently available therapies, and drugs targeting specific points on those pathways are already being tested on phase II studies, with promising results. Further studies focusing on the role of Th17 cells and their related molecules as biomarkers of diagnosis, disease activity, and response to specific therapies are warranted and may potentially lead to a more precise comprehension of MS and NMOSD, as well as more selective and effective therapies.

## Figures and Tables

**Table 1:** Hallmarks on the understanding of the role of the Th17 pathways in MS.

Finding	Reference
Increased IL-17 found in the blood and CSF of RRMS patients, especially during relapse	[43]

IL-17-producing T cells identified in EAE	[10]

Increased Th17 cells and IL-17 found in the brain of MS patients	[48]

IL-17 production correlates with MRI activity	[53]

Secukinumab (anti-IL-17A monoclonal antibody) reduces MRI lesions in a phase II clinical trial	[114]

**Table 2:** Hallmarks on the understanding of the role of the Th17 pathways in NMOSD.

Finding	Reference
Increased IL-17 found in the CSF of NMO patients	[60]

Increased Th17 cells found in the blood of NMO patients, especially during relapse	[69]

IL-17 and subsets of Th17 correlate with EDSS and relapse frequency in NMO	[54]

Pathogenicity of AQP4-specific T cells demonstrated in animal models	[65]

Pathogenicity of Th17-polarized AQP4-specific T cells demonstrated in animal models	[67]
